# 875. A Specialty on Fire: Burn Surgery’s Quiet Lead Toward Gender Equity

**DOI:** 10.1093/jbcr/irag033.352

**Published:** 2026-04-06

**Authors:** Eva Murphy, Kathleen S Romanowski, Anne L Lambert Wagner, Sharmila Dissanaike, Jeffrey E Carter, M Victoria P Miles, Colleen M Ryan

**Affiliations:** Burn Center at University Medical Center, New Orleans, LA; Shriners Children's - Northern California, Sacramento, CA; Vanderbilt University Medical Center, Nashville, TN; The University of New Mexico, Albuquerque, NM; University Medical Center Burn Center, Louisiana State University Health Sciences Center New Orleans, Louisiana; University Medical Center Burn Center, Louisiana State University Health Sciences Center, Department of Surgery, New Orleans, LA; Massachusetts General Hospital, Harvard Medical School, Boston, MA

## Abstract

**Introduction:**

Despite near gender parity in surgical residencies, women remain underrepresented across several high-acuity surgical subspecialties. While fields such as obstetrics/gynecology, breast surgery and endocrine surgery are now female-majority, more traditionally male-dominated disciplines, including trauma, orthopedics, and neurosurgery, continue to have disproportionately few female physicians. Burn surgery, which intersects with trauma, critical care, and reconstructive surgery, has received little attention in gender representation studies. With women now comprising over 43% of general surgery residents and more than 50% of medical students since 2017, the central question is shifting from whether the pipeline can support gender diversity to why some surgical subspecialties may already be seeing greater gains. This project assesses gender representation in burn surgery in comparison to other subspecialties and examines factors that may influence female recruitment and retention.

**Methods:**

We conducted a retrospective cross-sectional study of national burn surgeon workforce data (2019–2024) using ABA membership records. Gender composition was assessed annually and aggregated over five years. Published data from other surgical specialties were reviewed for comparison. Two surveys were developed: 1) a quantitative survey for fellowship program directors assessing gender composition of applicants and matriculants over five cycles; and 2) a mixed-methods survey for female burn surgeons exploring motivations, workplace culture, and gender-related challenges. Survey distribution is underway; results will be analyzed using descriptive and thematic analysis.

**Results:**

From 2019–2024, women comprised 36% (148/404) of practicing burn surgeons, substantially higher than in most surgical specialties. While year-over-year change was minimal, literature review confirms lower female representation (< 25%) across comparable high-acuity fields. Pending survey results will offer further insight into training culture, mentorship, and structural influences on this trend.

**Conclusions:**

Burn surgery appears to demonstrate one of the highest rates of female representation among male-dominated surgical specialties. These findings highlight the importance of exploring field-specific factors, such as culture, mentorship, or lifestyle, that may influence female recruitment and retention. Ongoing survey analysis will provide further insight into these dynamics and may inform strategies to support gender diversity across other surgical disciplines.

**Applicability of Research to Practice:**

This research offers data-driven insight to inform recruitment strategies, workforce development, and gender equity efforts in surgical education.

**Funding for the study:**

N/A.

